# Heterogeneous ozone effects on the DNA methylome of bronchial cells observed in a crossover study

**DOI:** 10.1038/s41598-020-72068-6

**Published:** 2020-09-25

**Authors:** M.-A. C. Bind, D. B. Rubin, A. Cardenas, R. Dhingra, C. Ward-Caviness, Z. Liu, J. Mirowsky, J. D. Schwartz, D. Diaz-Sanchez, R. B. Devlin

**Affiliations:** 1 Department of Statistics, Faculty of Arts and Sciences, Harvard University, Cambridge, MA USA; 2 Yau Center for Mathematical Sciences, Tsinghua University, Beijing, China; 3 Department of Statistical Science, Fox School of Business, Temple University, Philadelphia, PA USA; 4 Department of Environmental Health Sciences, UC Berkeley School of Public Health, Berkeley, CA USA; 5 Department of Environmental Sciences and Engineering, UNC Gillings School of Global Public Health, Chapel Hill, NC USA; 6 Environmental Public Health Division, NHEERL, US Environmental Protection Agency, Research Triangle Park, NC USA; 7 Department of Statistics and Actuarial Sciences, University of Hong Kong, Pok Fu Lam, Hong Kong; 8 Department of Chemistry, SUNY College of Environmental Science and Forestry, Syracuse, NY USA; 9 Department of Environmental Health, Harvard School of Public Health, Boston, MA USA

**Keywords:** Environmental sciences, Molecular medicine

## Abstract

We used a randomized crossover experiment to estimate the effects of ozone (vs. clean air) exposure on genome-wide DNA methylation of target bronchial epithelial cells, using 17 volunteers, each randomly exposed on two separated occasions to clean air or 0.3-ppm ozone for two hours. Twenty-four hours after exposure, participants underwent bronchoscopy to collect epithelial cells whose DNA methylation was measured using the Illumina 450 K platform. We performed global and regional tests examining the ozone versus clean air effect on the DNA methylome and calculated Fisher-exact *p*-values for a series of univariate tests. We found little evidence of an overall effect of ozone on the DNA methylome but some suggestive changes in *PLSCR1*, *HCAR1*, and *LINC00336* DNA methylation after ozone exposure relative to clean air. We observed some participant-to-participant heterogeneity in ozone responses.

## Introduction

Ozone is a ubiquitous air pollutant that has been studied more extensively than perhaps any other environmental toxicant. Both short-term^1,2^ and long-term^3,4^ exposures have been linked with increased mortality and morbidity^5,6^. Numerous randomized controlled human exposure studies have shown that ozone exposure causes decrements in lung function and increased pulmonary inflammation (reviewed in US EPA air quality criteria for ozone and related photochemical oxidants) with considerable heterogeneity across people, and a randomized study has described cardiovascular changes in humans exposed to ozone^7^. Multiple studies have reported ozone-induced changes in pro-inflammatory cytokines in the lung of humans exposed to ozone^7,8^, as well as changes in cytokine mRNA expression in bronchoalveolar lavage cells obtained following exposure of humans to ozone^9^. In addition, ozone has been shown to alter upstream mitogen-activated protein (MAP) kinase pathways that control cytokine gene expression^10,11^.

It has been proposed that adverse health effects caused by exposure to air pollutants may be mediated by epigenetic changes^12^. Exposure to air pollution has been associated in several recent non-randomized studies with epigenetic changes, especially DNA methylation. In one study^13^, exposure to traffic-related pollutants was associated with reduced lung function in elderly men and epigenetic changes in genes related to inflammation and immunity were proposed to modify the air pollution-lung function association. Increased air pollution was significantly associated with changes in DNA methylation of several genes, including those involved in inflammation^14^. A relationship between particulate air pollution and hypomethylation of the inducible nitric oxide synthesis gene was reported in a cohort of children^15^.

Because all these studies are non-randomized cohort studies in which changes in ambient pollutant levels are *associated* with epigenetic changes, it is difficult to claim a *causal* relationship between air pollutant exposure and epigenetic changes. Furthermore, these studies measured epigenetic changes in blood cells rather than the primary targets of inhaled pollutants (i.e., respiratory tract cells). To show a direct causal link between air pollutant exposure and biologic changes in humans, randomized controlled human exposure studies are considered to be the “gold standard”. A recent randomized controlled human exposure study measured DNA methylation changes in T helper cells found in blood and reported changes in methylation in genes involved in mitochondrial oxidative energy metabolism in subjects exposed to particulate air pollution^16^. Here, we report the results of a random exposure study in which human volunteers were randomly exposed to either 0.3-ppm ozone or clean air on two occasions separated by several weeks. Bronchoscopy was performed 24 h after each exposure to remove cells directly targeted by ozone; i.e., airway epithelial cells. DNA methylation following each person’s clean air exposure was then compared with DNA methylation following that person’s ozone exposure. Because of the small sample size of the experiment, we eschew standard asymptotic inference assuming Student’s t-distributions under the null hypothesis, and instead capitalize on exact randomization-based inference.

In our study, we go beyond the current state of knowledge on the effect of ozone on DNA methylation. In observational studies, we do not know whether the estimated *associations* between ozone and the health outcomes are confounded by other environmental exposures correlated with ozone (e.g., NO_2_, temperature). In this randomized setting, we can directly estimate, with uncertainties expressed by the results of the randomization tests, the effect of ozone on the DNA methylome of bronchial cells (another “gold standard” for the field of air pollution epigenetics).

## The crossover experiment

### Study participants

The study population and exposure design have been described in detail previously by Devlin et al.^7^, who estimated the causal effect of ozone versus clean air on the cardiovascular system. A total of seventeen healthy individuals (see their characteristics in Table 1) were recruited to participate in this study under a contract with Westat Corporation. Participants were excluded if they were smokers, pregnant, had any previous cardiopulmonary disease or allergies (as determined by their medical history and physical examination), or had a forced vital capacity (FVC) or forced expiratory volume in the first second of expiration (FEV_1_) of less than 80% predicted from their height and age. Prior to enrollment, all participants were informed of the study procedures and potential risks, and all provided a written informed consent. The consent forms and protocol were approved by the University of North Carolina School of Medicine and the US Environmental Protection Agency. The study was registered on ClinicalTrials.gov (NCT01492517).

**Table 1:** Characteristics of the seventeen participants.

	Min	25^th^	Mean	75^th^	Max
Age	21	23	25.4	27.5	33
Body mass index	17	23	25	27	29
Systolic blood pressure (mmHg)	106	113	121	130	136
Diastolic blood pressure (mmHg)	62	71	74	78	90
Heart rate (beats/min)	52	63	70	75	91
**Gender**					
Male			88%		
Female			12%		
**Race**					
White			88%		
Non-white			12%		

### Study design

We conducted a randomized, single-blind, crossover study in which each participant was exposed twice, for 2 h to clean air (i.e., PM, CO, NO_2_, SO_2_ concentrations were below detection limit) or 0.3-ppm ozone (2015 US 8-h ozone standard: 0.07 ppm). The exposures were only for 2 h, in contrast to the standard, which is for 8 h. The actual number of ozone molecules to which a person was exposed in this study was about what they would have breathed for 8 h at the current standard. The exposure sessions were separated by a minimum of 13 days in an attempt to avoid carry-over effects associated with the first exposure. The exposure chamber and pollutant generation system are described elsewhere^7^. During the 2-h exposure, participants alternated between exercising for 15 min on a cycle ergometer and resting for 15 min. Minute ventilation was measured during each exercise session, and exercise levels were adjusted to obtain a minute ventilation of approximately 25 L min^−1^ m^−2^ body surface area. All exposures were conducted at the same time of the day and on the same day of the week for all participants between July 2010 and June 2011. The meteorological conditions in Chapel Hill during the time of the study^17^ are presented in Table 2. As with any empirical studies, one can always object and find limitations in the study design: the volunteers were not housed and exposed to clean air or ozone for days, and some factors were not controlled (e.g., air pollution exposure preceding bronchoscopy).

**Table 2:** Meteorological conditions in Chapel Hill during the study time.

	Min	25^th^ percentile	Mean	Median	75^th^ percentile	Max
Ozone (ppm)	0.002	0.019	0.026	0.025	0.033	0.063
PM_2.5_ (µg/m^3^)	1.0	7.8	10.9	9.9	13.2	28.2
Temperature (°C)	− 7.6	7.3	15.1	15.6	23.3	32.8
Relative humidity (%)	26.5	57.9	67.9	70.0	78.4	96.3
Barometric pressure (hPa)	1,001.4	1,014.0	1,018.2	1,018.0	1,021.9	1,036.4

### Measurement and pre-processing of DNA methylation

Twenty-four hours following each exposure, the subjects underwent a research bronchoscopy with brush biopsy to obtain bronchial epithelial cells as described previously^18^. The cytology brushes containing epithelial cells tips were placed in a 1.5 mL tube with 200 µL Trizol Buffer (ThermoFisher Scientific), DNA extracted using the Gentra Puregene Buccal Cell Kit (Qiagen, Inc.). DNA samples were stored frozen at − 80 °C until analysis. DNA extracted from the bronchial epithelial cells was sent to a commercial laboratory (Expression Analysis, Durham, NC) for DNA methylation assessment using the Illumina HumanMethylation 450 K BeadChip platform. The extracted DNA samples were placed on four chips (see positions in Supplemental Fig. 1). Participants labelled 1, 3, 4, 6, 7, and 13 had both extracted DNA, i.e., after exposures to clean air and ozone, positioned on the same Chip.

We performed background correction using noob^19^, dye bias corrections, and corrected for probe design bias arising from Type I and Type II probes with the Beta-mixture quantile normalization method (*BMIQ*) method^20^. Although the modes of probe-type DNA methylation distributions were different, they were well aligned after the *BMIQ* procedure (see Supplemental Fig. 2). Methylation values are reported as the methylation beta value, i.e., the ratio of methylated probe signal intensities to methylated and unmethylated probe signal intensities. A small offset of 100 is added to the denominator to prevent inflated values when the sum of methylated and unmethylated probe signal intensities is low. After this pre-processing, the epigenomic analyses considered a set of 484,531 CpG sites, which we index by *k*.

### Epigenomic data

**Figure 1:**
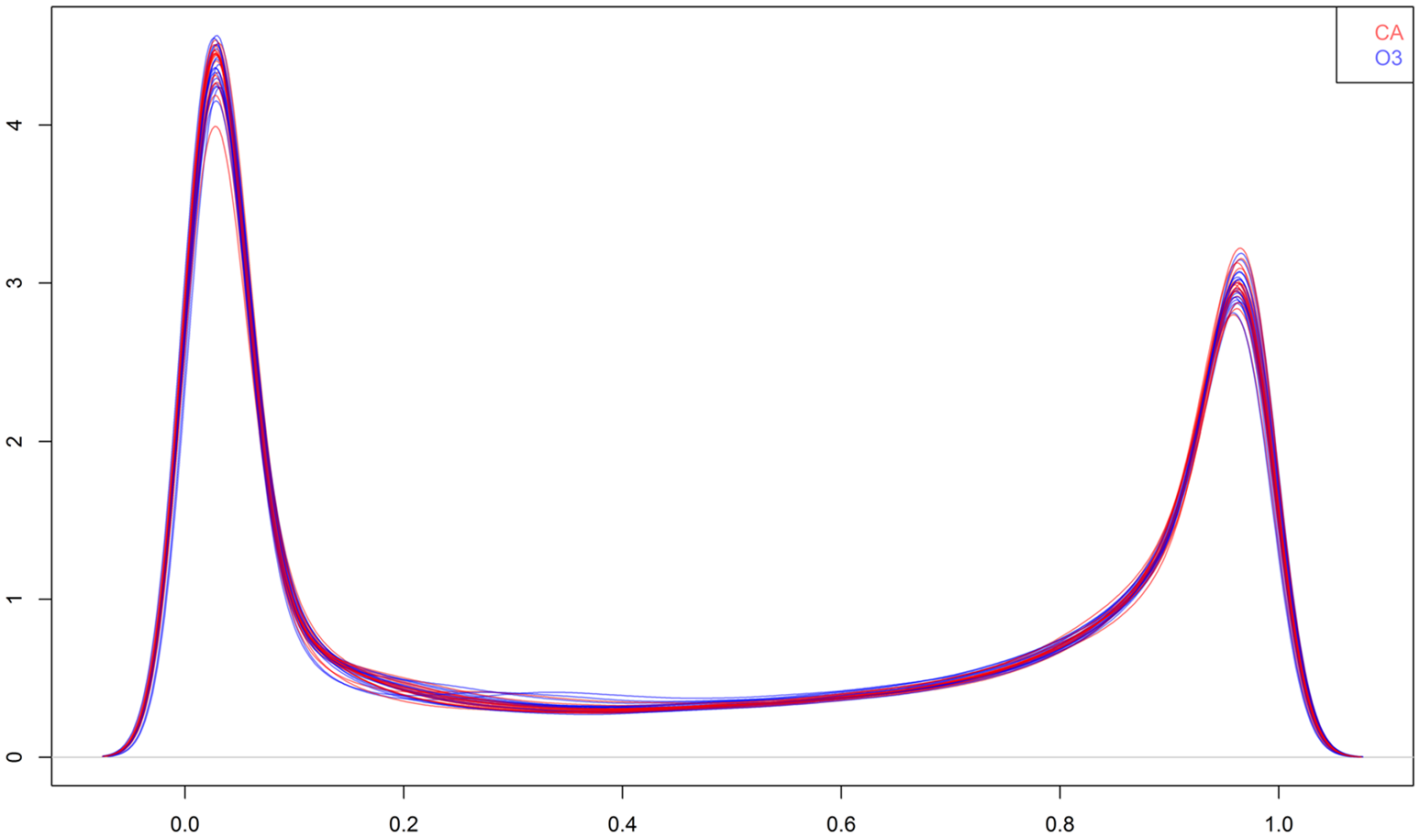
Participant distributions of DNA methylation after exposures to clean air (red) and ozone (blue).

The seventeen DNA methylome distributions observed after exposure to clean air, denoted by Y_i_^obs^(w_i_ = 0)—ignoring the crossover time period, and the seventeen ones observed after exposure to ozone, denoted by Y_i_^obs^(w_i_ = 1), are shown in Fig. 1. We denote the mean participant methylation at site *k* after clean air and ozone exposures by m_0,k_ = ($\frac {1} {17}$) Σ_i=1:17_ Y_i,k_^obs^(w_i_ = 0), and m_1,k_ = ($\frac {1} {17}$) Σ_i=1:17_ Y_i,k_^obs^(w_i_ = 1), respectively. The full set of K = 484,531 CpG sites includes locations near promoter regions. Although the empirical distribution of m_0,k_ was bimodal among the overall set of 484,531 CpGs, the empirical distribution of m_0,k_ was unimodal among 91,795 CpGs sites near promoter regions (see Supplemental Fig. 3), which supports the hypothesis that methylation sites near promoter regions tend to be hypomethylated compared to other CpG sites. Similar modes occur for the empirical participant median densities after exposure to clean air, median{Y_i,k_(w_i_ = 0)}, among both sets of methylation sites (results not shown). The boundaries displayed in Supplemental Fig. 3 (i.e., at levels 0.2%5mC and 0.8%5mC) define three levels of methylation (i.e., low, medium, and high) throughout the paper. These values were chosen based on the bimodal empirical distributions of the participant methylation mean and median among the overall set of CpGs after exposure to clean air.

Here, we perform statistical analyses on four sets of CpG sites: the epigenomic set of K = 484,531 CpG sites, and the sets of CpG sites with low, medium, and high methylation.

**Figure 2:**
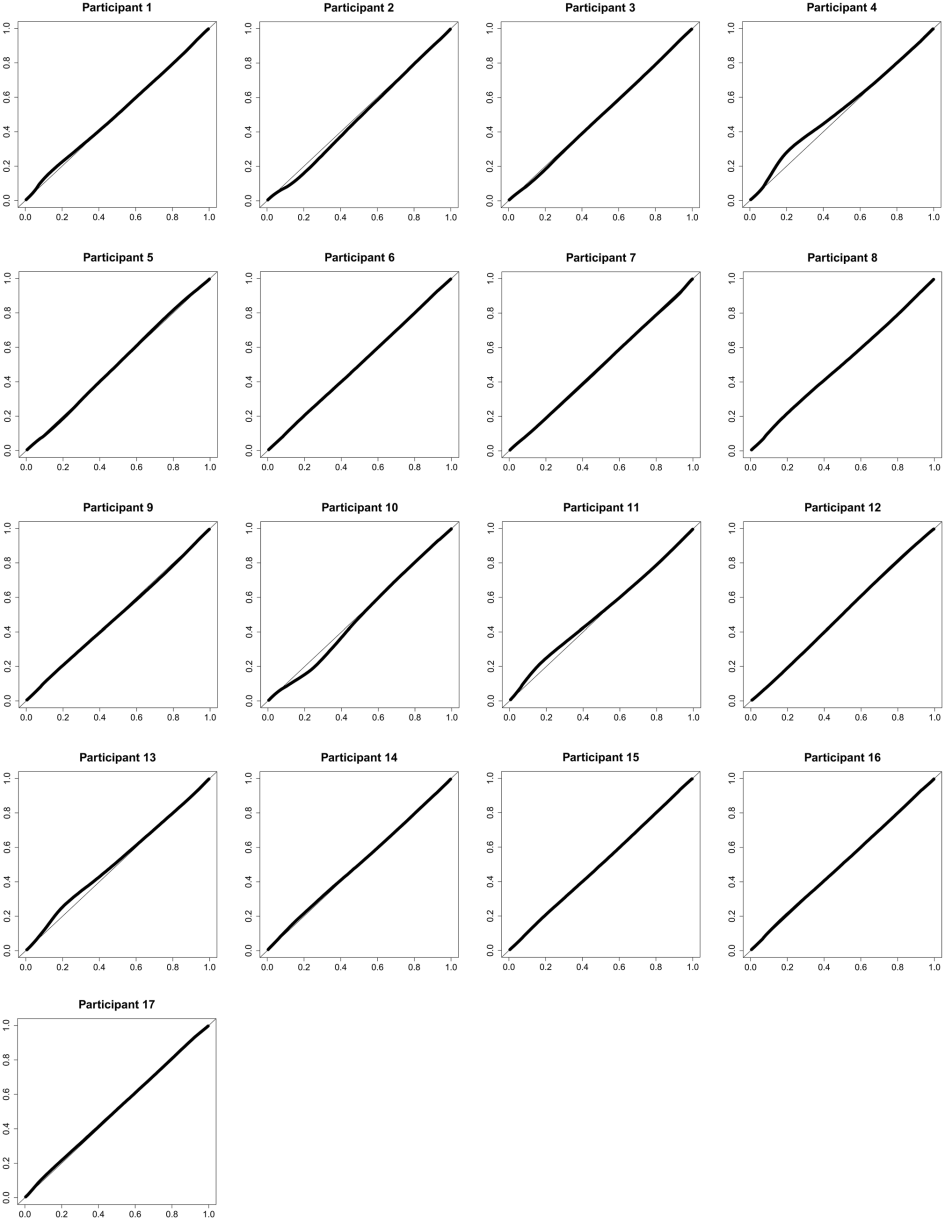
Participant-specific Q-Q plots for the empirical epigenomic participant methylation distributions after ozone versus after clean air.

Throughout, we use f(D) to denote the empirical distribution of the generic K-component vector D. The joint bivariate epigenomic distribution of: (1) the participant mean methylation distribution under clean air, and (2) the mean methylation distribution under ozone exposure, f(m_0,k_, m_1,k_)_k=1:484,531_, also suggests two modes, around low and high methylation levels (data not shown). The Q–Q plot between the empirical epigenomic participant mean methylation distribution after ozone versus after clean air, f(m_1,k_)_k=1:484,531_ versus f(m_0,k_)_k=1:484,531_ suggests that these two distributions are similar (results not shown). However, the participant-specific Q-Q plots show some deviations between the empirical epigenomic participant methylation distributions after ozone versus after clean air, Y_i_^obs^(w_i_=2) vs. Y_i_^obs^(w_i_=0), with the largest deviation for Participant *4* (Fig. 2).

## Methods

The overall statistical strategy we follow comprises five stages:

1. “Global analyses” assess the overall effect of ozone exposure on the DNA methylome,
2. “Regional analyses” assess whether ozone exposure changes DNA methylation of genomic regions,
3. “Local analyses” estimate the ozone effect on DNA methylation measured at each CpG site,
4. “Responsiveness analyses” assess whether the seventeen participants exhibit differential epigenomic responses to ozone exposure,
5. “Enrichment analyses” identify for each participant whether CpG sites are over-represented among the set of responsive CpG sites.

### Global tests for the O_3_ effect on the epigenome

#### Distributional tests

The mean participant methylations at site *k* after clean air and ozone exposures can be re-expressed as m_0,k_ = ($\frac {1} {N}$_CA-O3_) Σ_i,j:{wi,j=1}=0_ Y_i,j=1,k_(w_i,j=1_ = 0) + ($\frac {1} {N}$_O3-CA_) Σ_i,j:{wi,j=2}=0_ Y_i,j=2,k_(w_i,j=2_ = 0), and m_1,k_ = ($\frac {1} {N}$_CA-O3_) Σ_i,j:{wi,j=2}=1_ Y_i,j=2,k_(w_i,j=2_ = 1) + ($\frac {1} {N}$_O3-CA_) Σ_i,j:{wi,j=1}=1_ Y_i,j=1,k_(w_i,j=1_ = 1), where N_CA-O3_ and N_O3-CA_ are the number of participants who were first exposed to clean air (CA) or ozone (O_3_), respectively. The stochasticity of the exposure assignment mechanism resides in the order each participant is exposed to clean air or ozone, which, if we assume a Bernoulli (with probability 1/2) assignment mechanism, implies the existence of 2^17^ = 131,072 possible allocations. Using the Fisherian procedure to test the sharp null hypothesis (H_00_) stating for each participant *i*, for each CpG site *k*, Y_i,j=1,k_(w_i,j=1_ = 0) = Y_i,j=1,k_(w_i,j=1_ = 1) and Y_i,j=2,k_(w_i,j=1_ = 1, w_i,j=2_ = 0) = Y_i,j=2,k_(w_i,j=1_ = 0, w_i,j=2_ = 1), as described by Bind and Rubin^21^, we performed four randomization tests that compared the empirical participant mean methylation distributions under ozone versus clean air exposures for the four sets of CpG sites: (1) f(m_0,k_)_k=1:484,531_ versus f(m_1,k_)_k=1:484,531_, (2) f(m_0,k_)_k=1:197,754_ versus f(m_1,k_)_k=1:197,754_, (3) f(m_0,k_)_k=1:112,190_ versus f(m_1,k_)_k=1:112,190_, and (4) f(m_0,k_)_k=1:174,587_ versus f(m_1,k_)_k=1:174,587_. We chose the Kolmogorov–Smirnov (KS) distance as the test statistic to compare the two mean distributions of clean air versus ozone. Then, we calculated the KS distance for all possible allocations of the N × 2 matrix of exposure assignment w = (w_j=1_,w_j=2_) assuming the sharp null hypothesis (H_00_). Here, note that because the test statistic is symmetric, the number of unique values of KS distances under H_00_ is 2^17^/2 = 65,536 (assuming no ties).

#### Causal estimands and estimators

We define three causal estimands, none of which, as with all causal estimands, is directly observable:

1. the *i–j* unit causal effect (UCE) for site *k*, τ_i,j,k_, as the unobservable contrast τ_i,j,k_ = Y_i,j,k_(w_i,j_ = 1) − Y_i,j,k_(w_i,j_ = 0) (see Table 3),
2. the participant *i* (also unobservable) causal effect (PCE) for site *k*, τ_i,k_, as the average of the two unit-level causal effects, τ_i,1,k_, and τ_i,2,k_, i.e., τ_i,k_ = ($\frac {1} {2}$) (τ_i,1,k_ + τ_i,2,k_), and
3. the finite population, or average participant causal effect (APCE) for site *k*, τ_k_, as the average of the N individual-level causal effects τ_i,k_’s, τ_k_ = ($\frac {1} {17}$) Σ_i=1:17_ τ_i,k_; this is estimable under fewer assumptions than either UCE or PCE is.

**Table 3:** Science table of the crossover randomized experiment.

Participant *i*	Visit *j*	Epigenomic potential outcomes		Unit-level causal effect
		Y_i,j,k_(w_i,j_ = 0)	Y_i,j,k_(w_i,j_ = 1)	
1	1	Y_1,1,k_(w_1,1_ = 0)	Y_1,1,k_(w_1,1_ = 1)	τ_1,1,k_ = Y_1,1,k_(w_1,1_ = 1) − Y_1,1,k_(w_1,1_ = 0)
…	…	…	…	…
17	1	Y_17,1,k_(w_17,1_ = 0)	Y_17,1,k_(w_17,1_ = 1)	τ_17,1,k_ = Y_17,1,k_(w_17,1_ = 1) − Y_17,1,k_(w_17,1_ = 0)
1	2	Y_1,2,k_(w_1,2_ = 0)	Y_1,2,k_(w_1,2_ = 1)	τ_1,2,k_ = Y_1,2,k_(w_1,2_ = 1) − Y_1,2,k_(w_1,2_ = 0)
…	…	…	…	…
17	2	Y_17,2,k_(w_17,2_ = 0)	Y_17,2,k_(w_17,2_ = 1)	τ_17,2,k_ = Y_17,2,k_(w_17,2_ = 1) − Y_17,2,k_(w_17,2_ = 0)

The observed participant difference due to ozone versus clean air for site *k*, d_i,k_, is: Y_i,j=2,k_(w_i,j=2_ = 1) − Y_i,j=1,k_(w_i,j=1_ = 0) if participant *i* is exposed to clean air first (i: 1, …, N_CA-O3_), and Y_i,j=1,k_(w_i,j=2_ = 1) − Y_i,j=2,k_(w_i,j=2_ = 0) if participant *i* is exposed to ozone first (i: 1, …, N_O3-CA_) (see Table 4). We estimated the K = 484,531 average participant causal effects (APCEs) using d_k_, the average of the seventeen d_i,k_’s.

**Table 4:** Observed table of the crossover randomized experiment.

Participant *i*	Visit *j*	w_i,j_	Epigenomic potential outcomes		Observed participant difference d_i,k_
			Y_i,j,k_(w_i,j_ = 0)	Y_i,j,k_(w_i,j_ = 1)	
1	1	w_1,1_ = 0	Y_1,1,k_(w_1,1_ = 0)	?	d_1,k_ = Y_1,2,k_(w_1,2_ = 1) − Y_1,1,k_(w_1,1_ = 0)
1	2	w_1,2_ = 1	?	Y_1,2,k_(w_1,2_ = 1)	
…	…	…	…	…	…
17	1	w_17,1_ = 1	?	Y_17,1,k_(w_17,1_ = 1)	d_17,k_ = Y_17,1,k_(w_17,1_ = 1) − Y_17,2,k_(w_17,2_ = 0)
17	2	w_17,2_ = 0	Y_17,2,k_(w_17,2_ = 0)	?	

#### Average participant causal effects (APCEs) along the epigenome

We consider the marginal average participant causal effect (APCE) along the entire epigenome, and three conditional APCEs defined by boundaries of mean participant methylation at site k after clean air exposure, m_0,k_: low methylation; m_0,k_ ≤ 0.2%5mC; medium methylation, 0.2%5mC < m_0,k_ < 0.8%5mC; and high methylation, 0.8%5mC ≤ m_0,k_ ≤ 0.2%5mC).

In particular, f(τ) denotes the density of the K(= 484,531)-dimension vector of APCEs. Slightly abusing notation, we write τ = (τ_low_, τ_med_, τ_high_) as a permutation of τ such that:

1. f(τ_low_) is the density of the k_low_(= 197,754)-dimension vector of APCEs such that m_0,k_ ≤ 0.2%5mC,
2. f(τ_med_) is the density of the k_med_(= 112,190)-dimension vector of APCEs such that 0.2%5mC < m_0,k_ < 0.8%5mC,
3. f(τ_high_) is the density of the k_high_(= 174,587)-dimension vector of APCEs such that 0.8%5mC ≤ m_0,k_.

We examined the characteristics of the empirical distributions of the APCEs for all sites, that is, f(τ), and for the three subsets of CpG sites (low, medium, and high), that is, (1) f(τ_low_), (2) f(τ_med_), and (3) f(τ_high_).

### Regional epigenomic analyses

#### Comb-p

To identify potential differentially methylated regions (DMRs), we used *Comb-p* (Python), which groups neighboring Fisher-exact *p*-values that are small and weighted according to their observed autocorrelation^22^. We calculate the univariate Fisher-exact *p*-values based on the standard paired statistic and on the 2$\frac {17} {2}$ possible randomizations:

$$\begin{aligned} &{\text{T}}_{{{\text{k}},{\text{ paired}}}} = \left| {\left( {\frac{1}{17}} \right) \, \Sigma_{{{\text{i}} = {1}:{17}}} {\text{d}}_{{{\text{i}},{\text{k}}}} } \right|/\left[ {{\text{ s}}_{{\text{D}}} /{ 17}^{{{1}/{2}}} } \right], \hfill \\ &{\text{where s}}_{{\text{D}}}^{{2}} = \, \left[ {{1}/{16}} \right] \, \left[ {\Sigma_{{{\text{i}} = {1}:{17}}} \left( {{\text{d}}_{{{\text{i}},{\text{k}}}} - \, \left( {{1}/{17}} \right) \, \Sigma_{{{\text{i}} = {1}:{17}}} {\text{d}}_{{{\text{i}},{\text{k}}}} } \right)^{{2}} } \right]. \hfill \\ \end{aligned}$$

We use these Fisher-exact *p*-values to identify DMRs associated with ozone exposure with a minimum distance of 500 base pairs and a required *p*-value of 10^−3^ to start a region. For each CpG site within regions suggested by the *Comb-p* analysis, we report the estimated APCE and the associated Fisher-exact *p*-value.

#### DMRcate

We also examined averaged regional DNA methylation changes after ozone exposure using the R Bioconductor package *DMRcate*^23^. Briefly, this algorithm for regional DNA methylation analyses fits linear regression models via *limma* for each CpG and subsequently applies a Gaussian kernel smoothing function to test-statistics grouping significant probes based on genomic proximity. We modeled the mean DNA methylation difference between the clean air and ozone exposure at each CpG with an intercept only. For the discovery of candidate regions, given the multiple comparison issue, we chose the 10^−6^ threshold for statistical significance for the asymptotic, meaningless, p-values provided by *limma*.

### Local analysis of the average participant causal effects (APCEs) and associated meaningful *p*-values

For each CpG *k*, we compared the participant methylation mean after clean air exposure (m_0,k_) versus the participant methylation mean after ozone exposure (m_1,k_) using univariate Fisher-exact two-sided tests, similarly as in Bind and Rubin^21^. We then constructed Q–Q plots of observed versus “expected” *p*-values, Manhattan^24^ and Volcano^25^ plots, i.e.,

1. quantile–quantile of observed − log_10_(*p*-value) versus “expected” − log_10_(*p*-value),
− log_10_(*p*-value) versus chromosome number, and,
− log_10_(*p*-value) versus APCEs, respectively.

We compared (2) and (3) plots for *p*-values calculated with the Fisher-exact and approximating asymptotic paired Student’s tests.

### “*Epigenomic responsiveness*” to ozone

We estimated whether each participant *i* “*responded*” to ozone (with respect to the epigenome) by calculating the Cook’s distance^26^ of the estimated individual causal effect for the 484,531 CpG sites. Participant *i* was defined as an ozone “*epigenomic responder*” at CpG *k* if the Cook’s distance of its estimated individual causal effect was greater than four times the mean of the Cook’s distances of the estimated individual causal effects across the 17 participants. For each participant, we compare “*responsiveness*” counts, which are bounded by 484,531, the total number of CpG sites. As sensitivity analyses, we also increased the “*epigenomic responsiveness*” threshold from four to five and six times the mean of the Cook’s distances.

### Enrichment analyses for “*responsive*” CpGs

Based on the Cook’s distances described in the previous section, we identified (1) the individual(s) with a large number of responsive CpGs and describe them as “most-extreme responder(s)”, and (2) the individual(s) with a low number of responsive CpGs, the “least responder(s)”. We compared them in enrichment analyses. For each responder, we took the list of CpGs that were responsive in that individual, then removed from that list all the CpGs that were also responsive in the non-responders and performed enrichment analysis on the final gene list. Thus, enrichment analyses were performed for each responder based on an inter-individual comparison of responsive CpGs for responder(s) versus non-responder(s). We examined enrichment for Kyoto Encyclopedia of Genes and Genomes (KEGG) pathways using the *missMethyl* R package while adjusting for varying gene size to avoid bias from large genes being overrepresented among the CpGs. We used the corrected *p*-values using the false discovery rate (FDR) to identify possible enriched pathways at a significance level of 0.05.

All methods were performed in accordance with the relevant guidelines and regulations.

## Results

### Global tests of the O_3_ effect on the epigenome

According to the four randomization tests comparing the participant mean methylation distributions under clean air versus ozone, we found some evidence against one or two of the four sharp null hypotheses (0.07 ≤ *p*-values ≤ 0.21, Table 5). The randomization distributions assuming the sharp null hypothesis are presented in Supplemental Fig. 4, suggesting that the asymptotic KS *p*-values, all less than 10^−10^, are deceptive.

**Table 5:** Global Fisher-exact tests.

Null hypothesis: no effect of ozone versus clean air	Kolmogorov–Smirnov distance	Fisher-exact *p*-value
f(m_0,k_)_1:484,531_ versus f(m_1,k_)_1:484,531_	D = 0.0083	0.21
f(m_0,k_)_k=1:197,754_ versus f(m_1,k_)_k=1:197,754_	D = 0.0204	0.17
f(m_0,k_)_k=1:112,190_ versus f(m_1,k_)_k=1:112,190_	D = 0.0138	0.12
f(m_0,k_)_k=1:174,587_ versus f(m_1,k_)_k=1:174,587_	D = 0.0124	0.07

The asymptotic Kolmogorov-tests led to highly significant *p*-values (< 10^−10^) compared to the ones obtained using the actual randomization of the crossover experiment.

**Figure 3:**
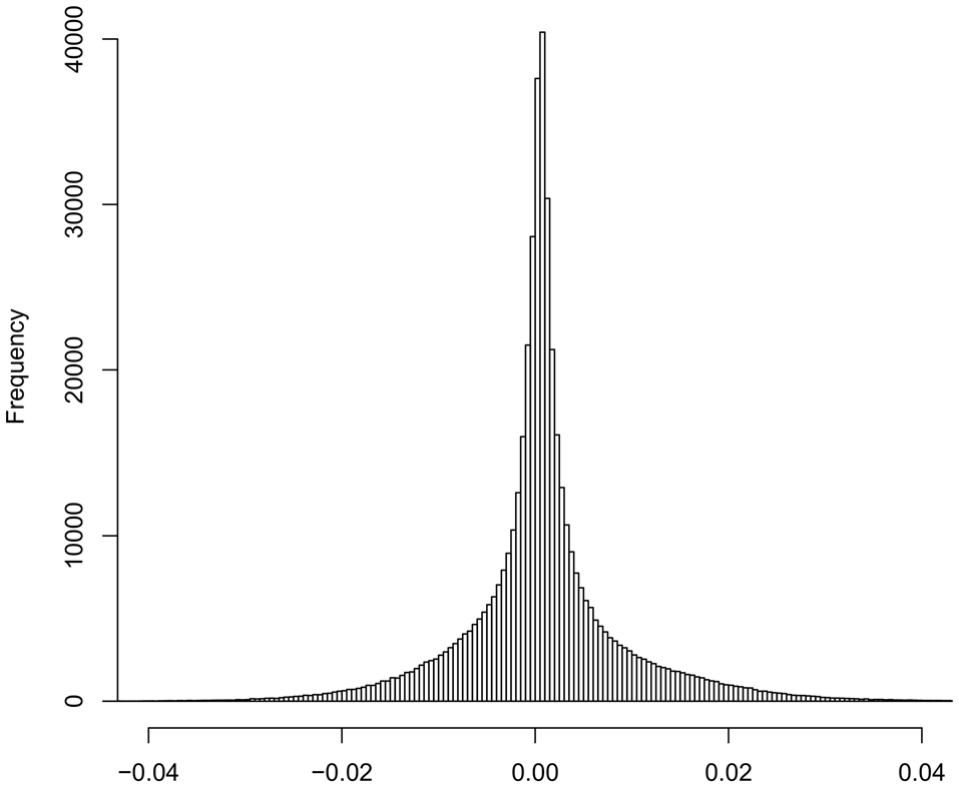
Empirical distribution of the estimated average participant causal effects (K = 484,531 APCEs).

**Figure 4:**
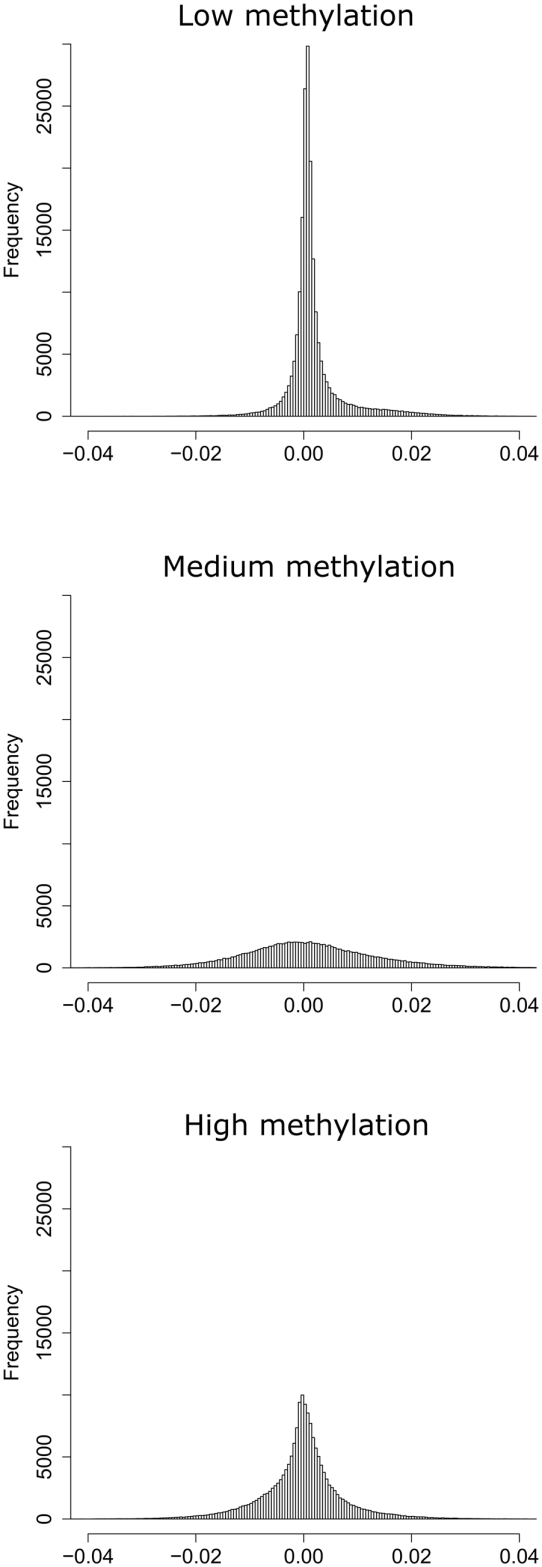
Empirical distributions of the estimated average participant causal effects (APCEs) for low, medium, and high DNA methylation sites (i.e., low: [0.0%5mC; 0.2%5mC], medium: ]0.2%5mC–0.8%5mC[, and high: [0.8%5mC; 1.0%5mC]).

The histogram of the estimated average participant causal effects (APCEs) for all loci, f(d_k_)_k=1:484,531_, appears to follow roughly a double-exponential distribution (Fig. 3). Similarly, (1) for loci with low methylation (≤ 0.2%5mC), f(d_k,low_)_k=1:197,754_, appears to follow roughly a double-exponential distribution (Fig. 4, top panel), (2) for loci with medium methylation (> 0.2%5mC and ≤ 0.8%5mC), f(d_k,med_)_k=1:112,190_, appears to follow a bell-shaped distribution (Fig. 4, middle panel), and (3) for loci with high methylation (> 0.8%5mC), f(d_k,high_)_k=1:174,587_, appears to follow a roughly double-exponential distribution (Fig. 4, bottom panel). The characteristics of these distributions for different boundaries (e.g., at 0.1%5mC and 0.9%5mC or at 0.3%5mC and 0.7%5mC) do not alter these conclusions (results not shown). We chose the exponential distribution to model the empirical distributions across CpG sites of the: (1) positive and (2) negative estimated APCEs and estimated the rate using the “*fitdist*” R function. We constructed a Q–Q plot between random draws from an exponential distribution (with estimated rate equal to 190) and the positive APCEs across 484,531 CpG sites (Supplemental Fig. 5, left graph). We proceed similarly for the negative APCEs and constructed a Q–Q plot between random draws from an exponential distribution (with estimated rate equal to 186) and the absolute value of the negative APCEs across 484,531 CpG sites (Supplemental Fig. 5, right graph). We observed that the negative APCEs can be fit with exponential distributions, although it appears more complex to find the approximating distributions for the positive APCEs.

### Regional tests of the ozone effect on the epigenome

#### Comb-p

Although we found limited evidence of a global effect of ozone on the epigenome, using regional analyses, we observed differences in DNA methylation of two CpGs in the *Phospholipid Scramblase 1* (*PLSCR1)* gene, nine CpGs in the *Hydroxycarboxylic Acid Receptor 1* (*HCAR1)* gene, and eight CpGs in the *Long Intergenic Non-Protein Coding RNA 336* (*LINC00336)* gene after exposure to ozone compared to clean air (Table 6).

**Table 6:** Differentially methylated regions (DMRs) and associated CpGs found using Comb-p analysis.

Genomic location	Number of CpGs	Nearby Gene	CpG site	Estimated average participant causal effect (APCE)	Fisher exact *p*-value^a^
*Chromosome 3 (146262175–146,262,422)*	*2*	*PLSCR1* *(software p-value* = *0.0026)*	cg10679755	0.0252 − 0.0212 = 0.0040	0.0187
			cg23486067	0.0258 − 0.0194 = 0.0064	0.0154
*Chromosome 12 (123215011–123215554)*	*9*	*GPR81* *alias HCAR1* *(software p-value* = *0.0019)*	cg22534509	0.3834 − 0.3560 = 0.0274	0.0135
			cg05290737	0.3522 − 0.3328 = 0.0194	0.0153
			cg22972858	0.4241 − 0.4025 = 0.0216	0.0068
			cg20566840	0.4294 − 0.3998 = 0.0296	0.0428
			cg01306688	0.2559 − 0.2356 = 0.0203	0.1680
			cg19975916	0.2122 − 0.1987 = 0.0135	0.2715
			cg19328828	0.1503 − 0.1251 = 0.0252	0.1268
			cg23505823	0.3863 − 0.3537 = 0.0327	0.0499
			cg13702536	0.4898 − 0.4543 = 0.0355	0.0138
*Chromosome 6 (33560954–33561450)*	*8*	*C6orf227* *Alias LINC00336* *(software p-value* = *0.0084)*	cg04329454	0.2349 − 0.2018 = 0.0331	0.0039
			cg01392313	0.1600 − 0.1315 = 0.0284	0.0015
			cg06289138	0.1711 − 0.1535 = 0.0176	0.0949
			cg08301503	0.1479 − 0.1276 = 0.0203	0.0015
			cg07873320	0.1926 − 0.1852 = 0.0073	0.1752
			cg05602975	0.1069 − 0.0978 = 0.0091	0.0877
			cg19869469	0.1632 − 0.1454 = 0.0178	0.0007
			cg00536532	0.6582 − 0.6683 = − 0.0101	0.2623

^a^Based on 2^17^ replications.

#### DMRcate

The genes detected by the *Comb-p* approach were also detected by the *limma*-based approach *DMRcate*: six CpGs in the *PLSCR1* gene, four CpGs in the *HCAR1* gene, and four CpGs in the *LINC00336* gene (Table 7).

**Table 7:** Differentially methylated regions (DMRs) and associated CpGs found using *DMRcate* with a threshold *p* = 10^−6^.

Genomic location	Number of CpGs	Nearby Gene	CpG site	Estimated average participant causal effect (APCE)	Fisher exact paired *p*-value^a^
*chromosome3* *(146261941–146262421)*	*6*	*PLSCR1*	cg05452836	0.0284 − 0.0250 = 0.0034	0.0083
			cg10679755	0.0252 − 0.0212 = 0.0040	0.0187
			cg12662193	0.0346 − 0.0257 = 0.0089	0.0004
			cg14795253	0.0287 − 0.0226 = 0.0061	0.0012
			cg15437043	0.2160 − 0.2143 = 0.0017	0.8154
			cg23486067	0.0258 − 0.0194 = 0.0064	0.0154
*Chromosome 12 (123215010–123215308)*	*7*	*HCAR1*	cg00357958	0.4270 − 0.4037 = 0.0234	0.0034
			cg01306688	0.2559 − 0.2356 = 0.0203	0.1680
			cg05290737	0.3522 − 0.3328 = 0.0194	0.0153
			cg19975916	0.2122 − 0.1987 = 0.0135	0.2715
			cg20566840	0.3998 − 0.4294 = 0.0296	0.0428
			cg22534509	0.3834 − 0.3560 = 0.0274	0.0135
			cg22972858	0.4241 − 0.4025 = 0.0216	0.0068
*Chromosome 6 (33560953–33561449)*	*9*	*LINC00336*	cg00536532	0.6582 − 0.6683 = − 0.0100	0.2623
			cg01392313	0.1600 − 0.1315 = 0.0284	0.0015
			cg04329454	0.2349 − 0.2018 = 0.0331	0.0039
			cg05602975	0.1069 − 0.0978 = 0.0091	0.0877
			cg06289138	0.1711 − 0.1535 = 0.0176	0.0949
			cg07873320	0.1926 − 0.1852 = 0.0073	0.1752
			cg08301503	0.1479 − 0.1276 = 0.0203	0.0015
			cg19735538	0.0763 − 0.0644 = 0.0118	0.1153
			cg19869469	0.1632 − 0.1454 = 0.0178	0.0007

^a^Based on 2^17^ replications.

### Local analysis of the average participant causal effects and associated p-values

Supplemental Fig. 6 displays the distribution of the univariate Fisher-exact *p*-values. Five CpG sites achieved the minimum *p*-value: *cg00605859*, *cg21964391*, *cg25265081*, *cg20129242*, and *cg11797346* (see Table 8 for a description of these CpG sites).

**Table 8:** Characteristics of CpG sites that achieve the minimum Fisher-exact *p*-value.

CpG site	Chromosome	Location	*UCSC gene*	UCSC region	Relation to CpG island	CpG island
*cg00605859*	15	73089234			Island	73089132–73089545
*cg21964391*	1	218253368				
*cg20129242*	6	75915754	*COL12A1*	TSS200	Island	75914705–75916387
*cg11797346*	8	55367611			Island	55366180–55367628
*cg25265081*	15	29346097	*APBA2*	Body		

**Figure 5:**
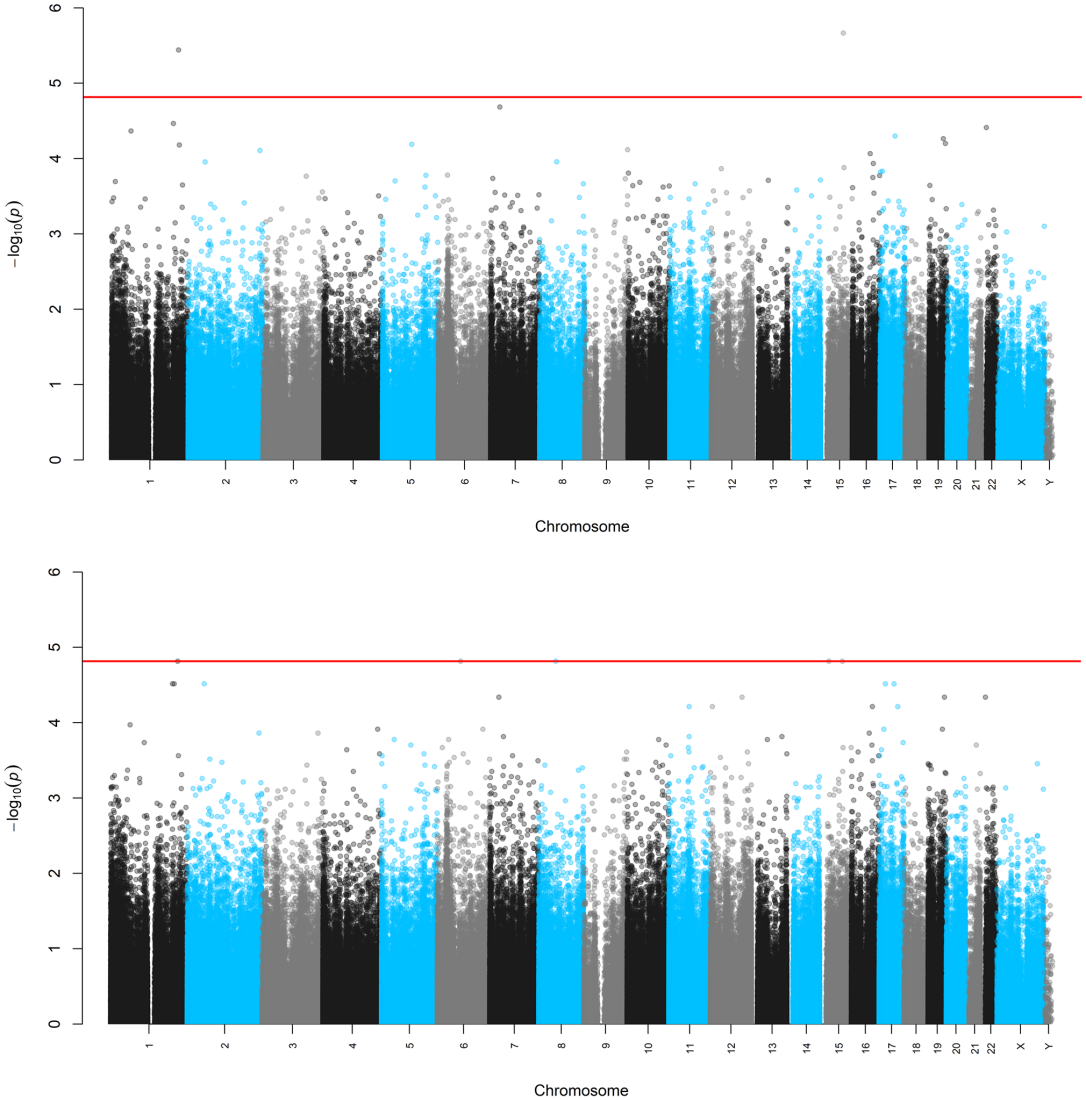
Manhattan plots of the univariate asymptotic *p*-values (top) and Fisher exact *p*-values (bottom).

**Figure 6:**
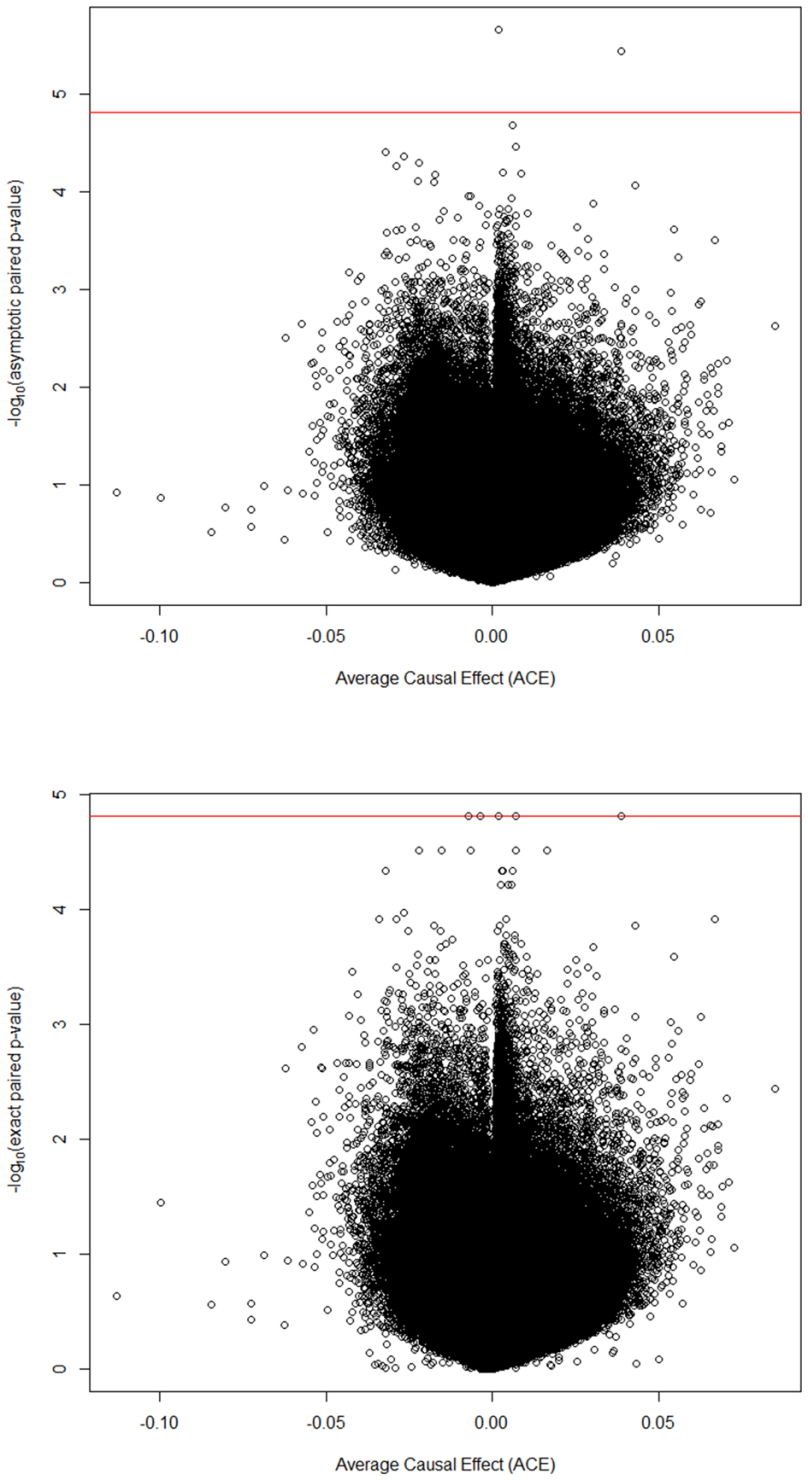
Volcano plots of the univariate *p*-values versus the average causal effect. Top plot: asymptotic *p*-values, bottom top: Fisher-exact *p*-values, and red line: maximum of − log_10_(*p*-value) that can be reached in this crossover experiment.

**Figure 7:**
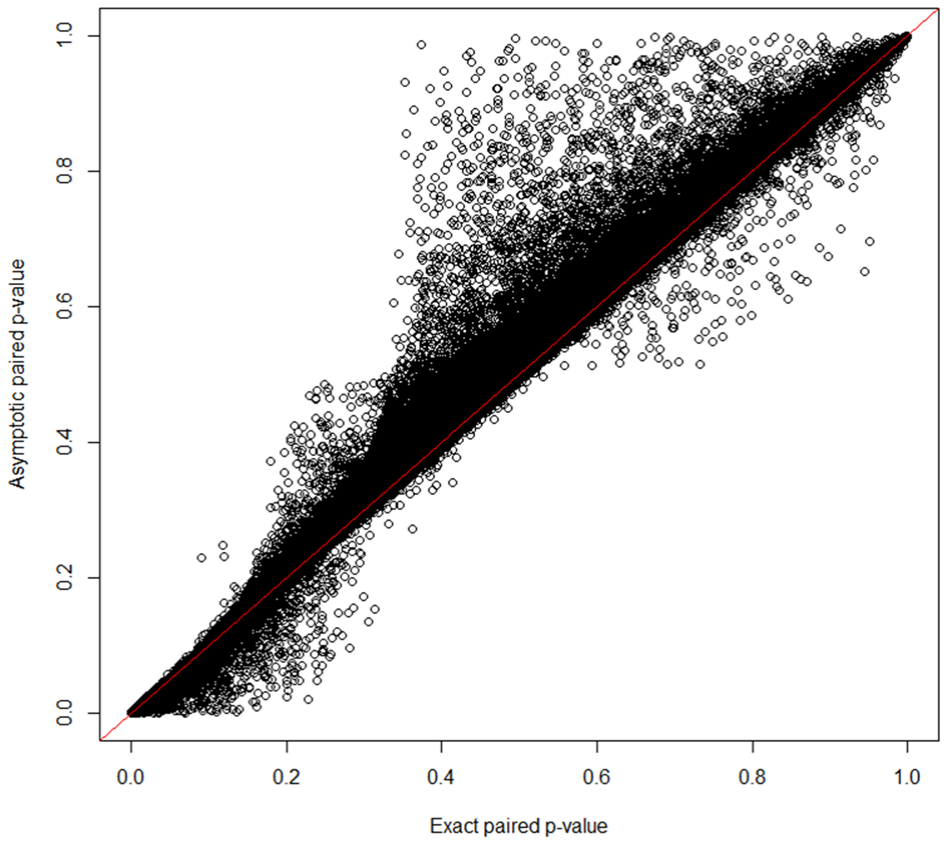
Asymptotic versus Fisher-exact (two-sided) *p*-values.

Even though the ratios of the median of the empirically observed distribution of the test statistic to the “expected” median are close to λ = 1, we observed some deviation of the observed − log_10_(*p*_value_) compared to the “expected” − log_10_(*p*_value_) (Supplemental Fig. 7). The deviation from the “expected” − log_10_(*p*_value_) could be explained by the CpG dependence, which is not taken into account for the construction of the “expected” − log_10_(*p*_value_). The Manhattan and Volcano plots are presented in Figs. 5 and 6, respectively. These plots were constructed using the Fisher-exact and approximating asymptotic *p*-values. We observed that the minimum possible *p*-value 2/2^17^ (or on the logarithmic scale, − log_10_(2/2^17^) = 4.8) was subceeded by some univariate asymptotic *p*-values. The Manhattan plot also indicates that the Bonferroni adjustment (with a threshold around 6 on the logarithmic scale) offers no power in this high-dimensional setting with small sample size. The asymptotic and Fisher-exact (two-sided) *p*-values do not agree in this crossover experiment (Fig. 7).

### “Responsiveness” to ozone with respect to the epigenome

**Figure 8:**
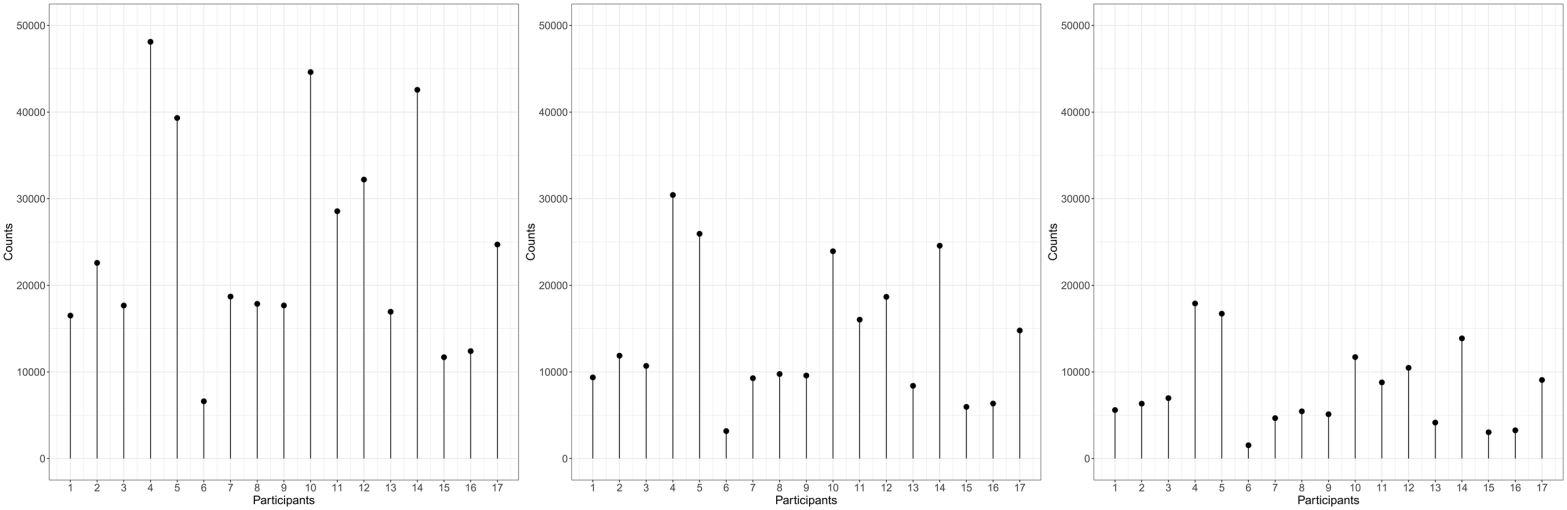
Responsiveness across subjects based on detection with Cook’s distance (threshold = 4, 5, and 6, from left to right).

We observed a difference in “epigenomic responsiveness” to ozone exposure: Participants *4*, *5*, *10*, and *14* had larger “epigenomic responses” to ozone than other participants, whereas Participant *6* had lower “epigenomic responses” (Fig. 8). That is, Participants *4*, *5*, *10*, and *14* were defined as “most-extreme responders” and Participant *6* as “least responder”.

### Enrichment analysis

When comparing the CpGs present among the most-extreme responders, there was no CpG that “*responded*” in all four of the responsive individuals. In contrast, at the pathway level, there was overlap between the pathways that were enriched among the *responsive* CpGs for Participants *4*, *5*, *10*, and *14*. Using Cook’s distance greater than four to define *responsive* CpGs, thirty pathways were enriched in the responsive CpGs for Individuals *4*, *5*, *10*, and *14*, but were not enriched in Individual *6*, the least responder (see Supplemental Table). Among these thirty pathways, twenty-one were enriched in all four responders and only two were enriched in just one responder. Pathways enriched in all four responders were linked to fatty acid and lipid metabolism, amino acid metabolism, and hormone/signaling molecule metabolism/biosynthesis.

## Discussion

We found some modest evidence for regional changes in the DNA methylome of bronchial cells after ozone exposure compared to clean air exposure. In contrast to observational studies examining the health^1,2^ or epigenetic^13,14^ effects of short-term exposure to ozone, these findings (1) are designed to be unconfounded, (2) are based on DNA methylation of respiratory tract cells, primary targets of inhaled ozone, and (3) do not assume approximating asymptotic distribution that can be inaccurate in small studies^21^. Airway epithelial cells present in where we obtained the biopsies consist of at least three cell types: ciliated cells, Clara cells (mucin secreting), and basal cells. In this study, we cannot determine the contribution of each cell type to the methylation results.

The thorough description of the characteristics of: (1) the DNA methylation distributions after exposures to clean air and ozone, (2) the empirical distributions of the average participant causal effects for the sites with low, medium, and high methylations, should motivate future statistical work. Traditional epigenome-wide analyses rely mostly on approximate asymptotic *p*-values, which is an issue in human experimental settings with small samples. Here, we perform statistical tests of the ozone effect on the DNA methylome and on site-specific tests using randomization-based inference to compute Fisher-exact *p*-values. Global tests did not reveal substantial changes in DNA methylome measured after ozone versus clean air, but there were some regional differences, as well as inter-individual differences. By comparing inter-individual differences, we were able to discover pathways that are potentially related to the “epigenomic responsiveness” to short-term ozone exposure.

To our knowledge, this is the first time that personalized epigenomic responsiveness is suggested. It is interesting that healthy participants are not responding homogeneously to short-term ozone exposure. New statistical methods should be developed to investigate *responsiveness*, in particular in high-dimensional settings. A study should further examine the estimated individual causal effects, especially whether the observed heterogeneity can be explained by background covariates such sex, age, race, DNA methylation under clean air. Future statistical research should also focus on the understanding of the high-dimensional characteristics of estimated average causal effects and should explore whether these distributions approximately follow double-exponential distributions, which has been used to model noise accumulation^27^. Other strategies that focus on computing accurate and robust *p*-value adjustments should also be explored^28^.

Using the number of CpGs per individual with substantial responsiveness to ozone based on their Cook’s distance, we were able to identify individuals who had an apparent excess, or deficit, of responsive CpGs. Four individuals were highly responsive to ozone from the perspective of DNA methylation loci changes, whereas one individual was “under-responsive”. Although there was no overlap in the individual CpGs among the responders, there was substantial overlap in the pathways enriched for altered CpGs among the most-extreme responders. Among “responsive” CpGs identified in the responders, 70% were present in all “ozone responders” and linked to pathways related to lipid and fatty acid metabolism and amino acid metabolism, among others. Long-chain fatty acids have been previously shown to be responsive to short-term exposure to NO_2_^29^. In the same participants of our study, fatty acid metabolism and amino acid metabolism were found to be enriched after ozone exposure compared to before the exposure^30^. Particulate matter-associated amino acids were also strongly associated with markers of inflammation and lung function^31^.

At the pathway level, there were three genes (i.e., *HCAR1*, *PLSCR1*, and *LINC00336*) that were uncovered in both the *DMRcate* and *Comb-p* analyses. *HCAR1* is located in an ozone exposure DMR on chromosome 12. *HCAR1* is an important lactic acid receptor molecule, which itself has a variety of known functions. Some functions are mediated by *HCAR1*, including inhibition of pro-inflammatory and cytotoxic responses. In the brain, *HCAR1* mediates angiogenesis^32^ and is related to wound healing^33^. These functions have not been examined in the lung but may be a key function linking *HCAR1* and ozone given the tissue damage induced by ozone exposure. *PLSCR1* is related to epidermal growth factor (EGF) and its receptor (EGFR) signaling pathway. This gene is closely linked with apoptosis and cellular growth. In a mouse model, cells with elevated *PLSCR1* expression showed an eightfold decrease in growth^34^. Cellular growth is an important component of epithelial-layer revival after exposure to ozone. *LINC00336* is a long non-coding RNA that is involved in ferroptosis (i.e., type of programmed cell death dependent on iron and characterized by the accumulation of lipid peroxides) in the lung^35^.

## Conclusion

Short-term exposure to ozone may alter the epigenome in young, healthy individuals who had epigenomes with variable responsiveness to an identical exposure to ozone. The most responsive individuals had several shared enriched pathways that mirrored effects of short-term exposure to ozone on fatty acid and amino acid metabolism as seen in metabolomics data from the same individuals and independent cohorts. Future studies are needed to further expand on the effects observed here and to integrate data to obtain a multiomic understanding of short-term ozone exposure effects.

## Supplementary information

Supplementary Information.

## References

[1] Bell ML, McDermott A, Zeger SL, Samet JM, Dominici F (2004). Ozone and short-term mortality in 95 US urban communities, 1987–2000. JAMA.

[2] Di Q (2017). Association of short-term exposure to air pollution with mortality in older adults. JAMA.

[3] Jerrett M (2009). Long-term ozone exposure and mortality. N. Engl. J. Med..

[4] Turner MC (2016). Long-term ozone exposure and mortality in a large prospective study. Am. J. Respir. Crit. Care Med..

[5] Urman R (2014). Associations of children's lung function with ambient air pollution: joint effects of regional and near-roadway pollutants. Thorax.

[6] McConnell R (2002). Asthma in exercising children exposed to ozone: a cohort study. Lancet.

[7] Devlin RB (2012). Controlled exposure of healthy young volunteers to ozone causes cardiovascular effects. Circulation.

[8] Alexis NE (2010). Low-level ozone exposure induces airways inflammation and modifies cell surface phenotypes in healthy humans. Inhal. Toxicol..

[9] Leroy P (2015). Inflammatory and repair pathways induced in human bronchoalveolar lavage cells with ozone inhalation. PLoS ONE.

[10] McCullough SD (2014). Ozone induces a proinflammatory response in primary human bronchial epithelial cells through mitogen-activated protein kinase activation without nuclear factor-kappaB activation. Am. J. Respir. Cell Mol. Biol..

[11] Yan Z (2016). Inflammatory cell signaling following exposures to particulate matter and ozone. Biochim. Biophys. Acta.

[12] Bollati V, Baccarelli A (2010). Environmental epigenetics. Heredity.

[13] Lepeule J (2014). Epigenetic influences on associations between air pollutants and lung function in elderly men: the normative aging study. Environ. Health Perspect..

[14] Bind MA (2014). Air pollution and gene-specific methylation in the normative aging study: association, effect modification, and mediation analysis. Epigenetics.

[15] Salam MT (2012). Genetic and epigenetic variations in inducible nitric oxide synthase promoter, particulate pollution, and exhaled nitric oxide levels in children. J. Allergy Clin. Immunol..

[16] Zhong J (2017). B vitamins attenuate the epigenetic effects of ambient fine particles in a pilot human intervention trial. Proc. Natl. Acad. Sci. USA.

[17] Mirowsky JE (2017). Ozone exposure is associated with acute changes in inflammation, fibrinolysis, and endothelial cell function in coronary artery disease patients. Environ. Health.

[18] Ghio AJ, Kim C, Devlin RB (2000). Concentrated ambient air particles induce mild pulmonary inflammation in healthy human volunteers. Am. J. Respir. Crit. Care Med..

[19] Triche TJ, Weisenberger DJ, Van Den Berg D, Laird PW, Siegmund KD (2013). Low-level processing of illumina infinium DNA methylation BeadArrays. Nucl. Acids Res..

[20] Teschendorff AE (2013). A beta-mixture quantile normalization method for correcting probe design bias in Illumina Infinium 450 k DNA methylation data. Bioinformatics.

[21] Bind MA, Rubin DB (2020). When possible, report Fisher-exact p-values and display the underlying null randomization distributions. Proc. Natl. Acad. Sci. USA.

[22] Pedersen BS, Schwartz DA, Yang IV, Kechris KJ (2012). Comb-p: software for combining, analyzing, grouping and correcting spatially correlated P-values. Bioinformatics.

[23] Peters TJ (2015). De novo identification of differentially methylated regions in the human genome. Epigenetics Chromatin.

[24] Gibson G (2010). Hints of hidden heritability in GWAS. Nat. Genet..

[25] Li W (2012). Volcano plots in analyzing differential expressions with mRNA microarrays. J. Bioinform. Comput. Biol..

[26] Cook RD (1977). Detection of influential observation in linear regression. Technometrics.

[27] Bao G, Schild D (2014). Fast and accurate fitting and filtering of noisy exponentials in legendre space. PLoS ONE.

[28] Zhang Y, Liu JS (2011). Fast and accurate approximation to significance tests in genome-wide association studies. J. Am. Stat. Assoc..

[29] Ward-Caviness CK (2016). Short-term NO2 exposure is associated with long-chain fatty acids in prospective cohorts from Augsburg, Germany: results from an analysis of 138 metabolites and three exposures. Int. J. Epidemiol..

[30] Cheng W (2018). Changes in metabolites present in lung-lining fluid following exposure of humans to ozone. Toxicol. Sci..

[31] Menni C (2015). Circulating levels of antioxidant vitamins correlate with better lung function and reduced exposure to ambient pollution. Am. J. Respir. Crit. Care Med..

[32] Morland C (2017). Exercise induces cerebral VEGF and angiogenesis via the lactate receptor HCAR1. Nat. Commun..

[33] Sun S, Li H, Chen J, Qian Q (2017). Lactic acid: no longer an inert and end-product of glycolysis. Physiology (Bethesda).

[34] Chawla-Sarkar M (2003). Apoptosis and interferons: role of interferon-stimulated genes as mediators of apoptosis. Apoptosis.

[35] Wang M (2019). Long noncoding RNA LINC00336 inhibits ferroptosis in lung cancer by functioning as a competing endogenous RNA. Cell Death Differ..

