# Supplementary material for: Heterogeneous ozone effects on the DNA methylome of bronchial cells observed in a crossover study
Source: Sci Rep. 2020 Sep 25;10:15739. doi: 10.1038/s41598-020-72068-6 (PMC7519112; doi:10.1038/s41598-020-72068-6)

**HETEROGENEOUS OZONE EFFECTS ON THE DNA METHYLOME OF BRONCHIAL CELLS OBSERVED IN A CROSSOVER STUDY**

M.-A. C. Bind^1^, D. Rubin^2,3^, A. Cardenas^4^, R. Dhingra^5^, C. Ward-Caviness^6^, Z. Liu^7^, J. Mirowsky^8^, J. D. Schwartz^9^, D. Diaz-Sanchez^6^, R. B. Devlin^6^

^1^ Department of Statistics, Faculty of Arts and Sciences, Harvard University, Cambridge, MA, USA

^2^ Yau Center for Mathematical Sciences, Tsinghua University, Beijing, China

^3^ Department of Statistical Science, Fox School of Business, Temple University, Philadelphia, PA, USA

^4^ Department of Environmental Health Sciences, UC Berkeley School of Public Health, Berkeley, CA, USA

^5^ Department of Environmental Sciences and Engineering, UNC Gillings School of Global Public Health, Chapel Hill, NC, USA

^6^ Environmental Public Health Division, NHEERL, US Environmental Protection Agency, Research Triangle Park, North Carolina, USA

^7^ Department of Statistics and Actuarial Sciences, University of Hong Kong, Hong Kong

^8^ Department of Chemistry, SUNY College of Environmental Science and Forestry, Syracuse, NY, USA

^9^ Department of Environmental Health, Harvard School of Public Health, Boston, MA, USA

**SUPPLEMENTARY MATERIALS**

**Supplemental Table**

**Supplemental Figure 1: Position of participants’ DNA on the four 450K Illumina chips**

**
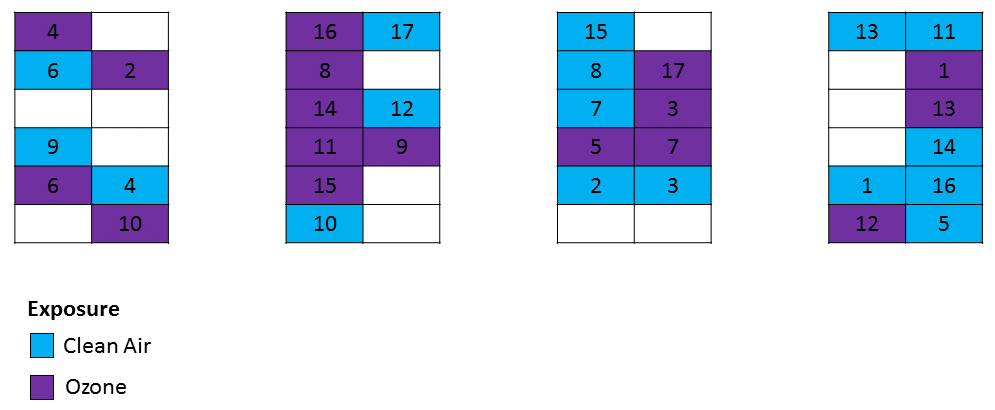
**

**Supplemental Figure 2: DNA methylome distributions across types of Infinium assays (red: type I and blue: type II) before (top) and after (bottom) the BMIQ procedure**

**
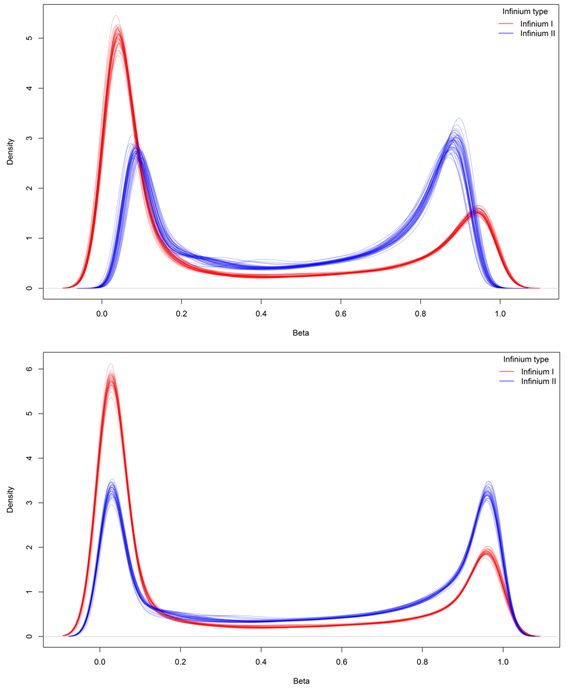
**

**Supplemental Figure 3: Empirical distribution of the participant mean methylation under clean air exposure (m_0,k_) across 484,531 CpG sites and across 91,795 CpG sites near promoter regions**


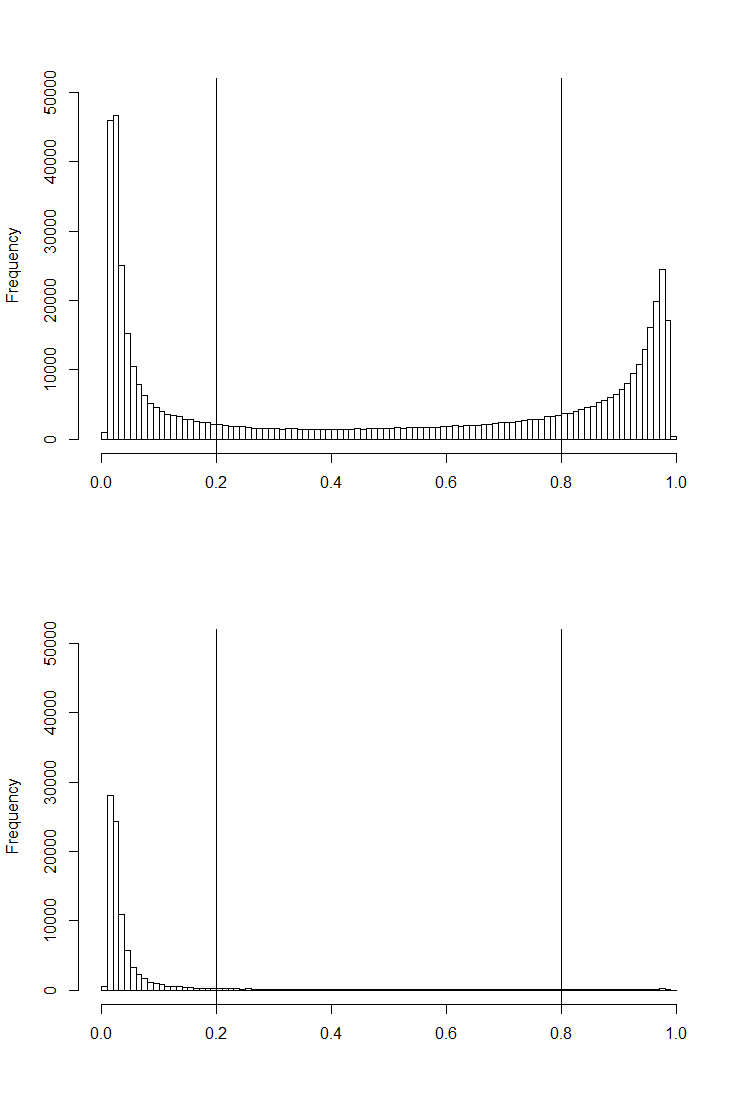


**Legend:**

450K: Low methylation: 197,754 sites; medium methylation: 112,190; high methylation: 174,587

Reduced: Low methylation: 83,181 sites; medium methylation: 6,459; high methylation: 2,155

**Supplemental Figure 4: Fisher-exact randomization tests comparing f(m_0,k_)_k=squeu1:484,531_ vs. f(m_1,k_)_k=1:484,531_ (top left), f(m_0,k_)_k=1:197,754_ vs. f(m_1,k_)_k=1:197,754_ (top right), f(m_0,k_)_k=1:112,190_ vs. f(m_1,k_)_k=1:112,190_ (bottom left), and f(m_0,k_)_k=1:174,587_ vs. f(m_1,k_)_k=1:174,587_ (bottom right).**


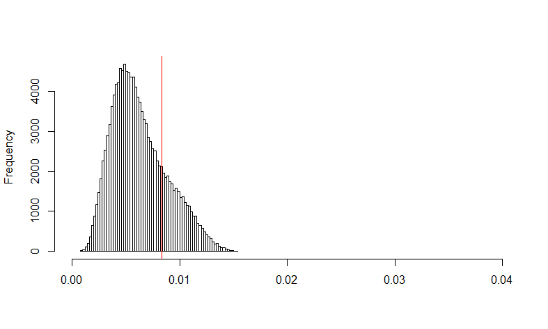

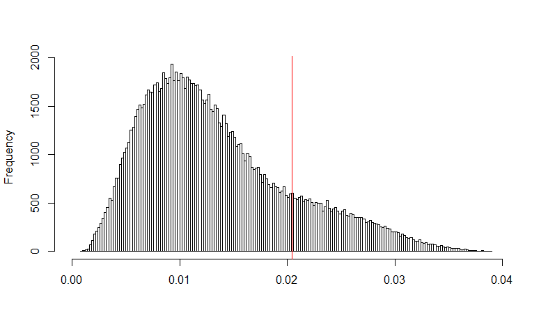


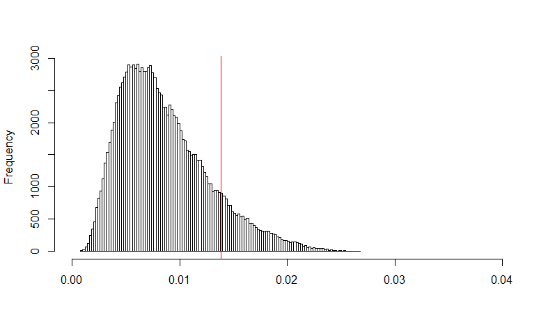

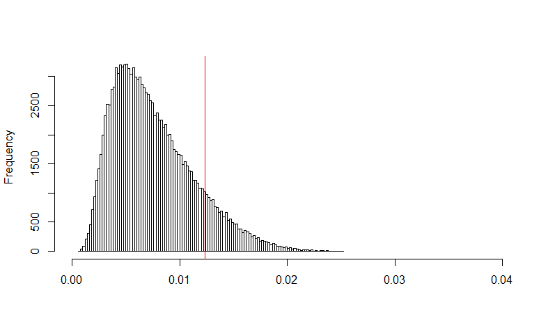


**Supplemental figure 5: Q-Q plots 1) between random draws from an exponential distribution (with rate 190) and the positive estimated APCEs (left graph), and 2) between random draws from an exponential distribution (with rate 186) and the absolute value of the negative estimated APCEs (right graph)**

**
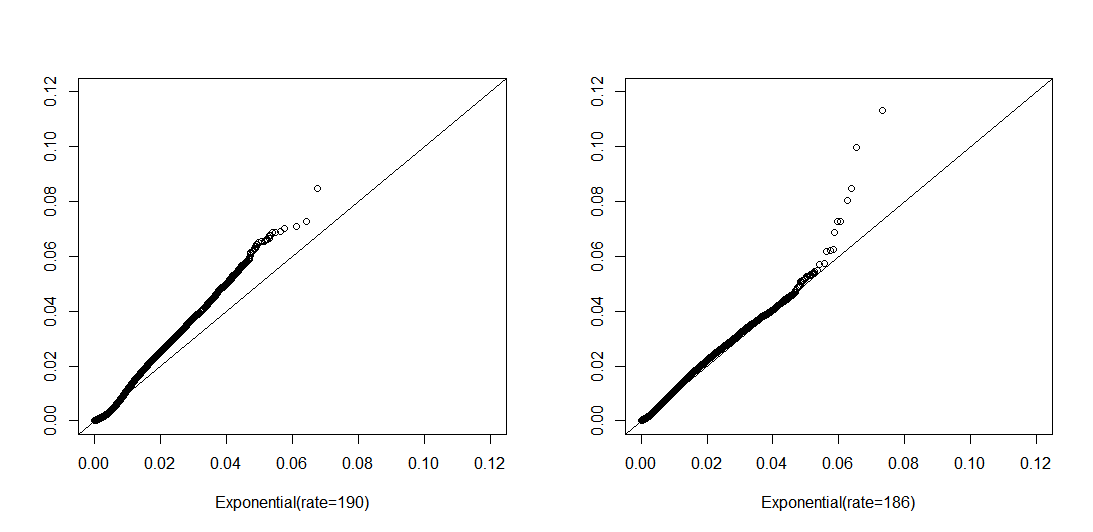
**

**Supplemental Figure 6: Distribution of the univariate Fisher-exact p-values**


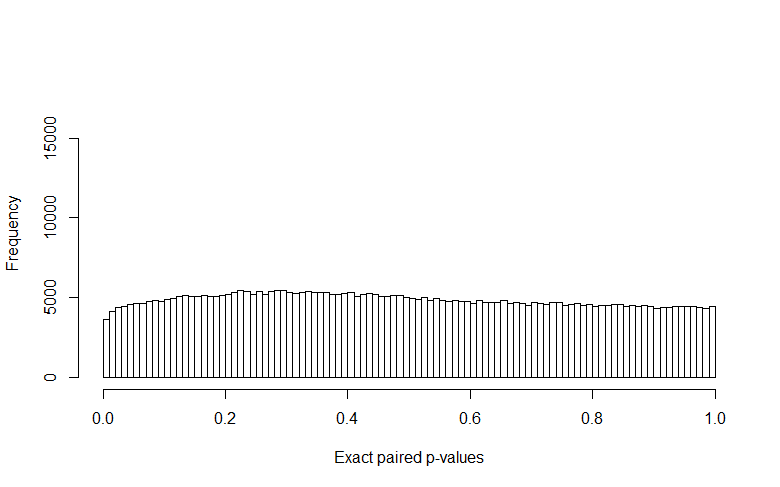


**Supplemental Figure 7: Observed -log_10_ Fisher-exact p-value versus “expected
 -log_10_ Fisher-exact p-value**


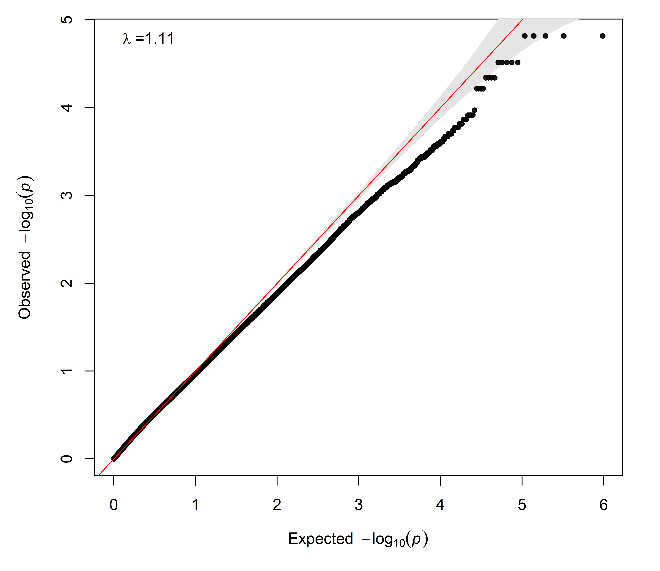

Supplement: Supplementary file 1 — Supplementary Information. [file 41598_2020_72068_MOESM1_ESM.docx]
